# The Additive Interaction between Body Mass Index and Hypertension Family History in Han and Yugur: The China National Health Survey (CNHS)

**DOI:** 10.1155/2019/8268573

**Published:** 2019-06-18

**Authors:** Chengdong Yu, Hongjun Zhao, Li Pan, Jia Zhang, Xiaoyang Wang, Lijun Chang, Ya Tuo, Jin'en Xi, Bin Liu, Ye Wang, Huiru Ren, Huijing He, Xiaolan Ren, Guangliang Shan

**Affiliations:** ^1^Department of Epidemiology and Statistics, Institute of Basic Medical Sciences, Chinese Academy of Medical Sciences and Department of Epidemiology and Statistics, School of Basic Medicine, Peking Union Medical College, Beijing 100005, China; ^2^Institute of Chronic and Noncommunicable Disease Control and Prevention, Gansu Provincial Centre for Disease Control and Prevention, Lanzhou 730000, China; ^3^Institute of Radiation Medicine, Chinese Academy of Medical Sciences, Peking Union Medical College, Tianjin 300000, China

## Abstract

**Objective:**

To estimate the additive interaction of body mass index (BMI) and family history of hypertension (FHH) on hypertension and explore whether the interaction could be influenced by behavioural risk factors.

**Methods:**

The cross-sectional data on 5791 participants were from the China National Health Survey in Gansu province in 2016. We assessed the additive interaction by calculating the relative excess risk due to interaction (RERI), the attributable proportion due to interaction (AP), and the synergy index (SI).

**Results:**

ORs for hypertension were highest in Han (13.52, 95%* CI*: 9.45 to 19.34) and Yugur (13.85, 95%* CI*: 8.48 to 22.63) with the combination of obesity and FHH. The interaction of BMI and FHH was significant in Han people, with the RERI, AP, and SI and their 95%* CIs* being 2.48 (1.13 to 3.82), 0.33 (0.19 to 0.47), and 1.61 (1.26 to 2.07) for overweight and FHH and 6.32 (1.91 to 10.73), 0.47 (0.27 to 0.67), and 2.02 (1.33 to 3.07) for obesity and FHH, respectively. The interaction of BMI and FHH was not significant in Yugur people. Adjustment for behavioural risk factors had little influence on the interactions, and risks of hypertension remained increased.

**Conclusions:**

BMI and FHH were associated with hypertension, and the interaction of BMI and FHH on hypertension was significant in Han but not in Yugur people. Behavioural risk factors had little influence on the associations and interactions. The exacerbation of hypertension risks by overweight or obesity in hypertension families deserves attention in weight control and community care.

## 1. Introduction

Hypertension is an important global public-health challenge due to high prevalence and concomitant risks of other cardiovascular and chronic kidney diseases [1–3]. In China, the age-standardized prevalence of hypertension increased by 1.4% per year over the period of 2002-2012 [4], and the prevalence of hypertension was 29.6% among Chinese adults in 2012 [5]. Moreover, deaths caused by high blood pressure accounted for 24.6% of all the deaths in 2010 in China [6]. As modifiable and nonmodifiable risk factors have a large contribution to hypertension, a better understanding of their interplay is needed to tackle this major public-health concern.

Both body mass index (BMI) and family history of hypertension (FHH) were regarded as two major risk factors for hypertension in behavioural and genetic-environmental aspects, respectively [7–16], but the impact of their cooccurrence is still little known. FHH is generally collected in clinical and epidemiological settings, as it contains both shared genetic and environmental factors associated with hypertension. Studies have shown that a FHH is associated with higher levels of both systolic and diastolic blood pressure and a higher risk of hypertension [7, 8]. The interactions between shared genes and environments lead to the aggregation of blood pressure within a family and result in physiological and biochemical processes that contribute to the increase of blood pressure [13]. It is estimated that 30% to 60% of the interindividual variation of blood pressure can be explained by genetic factors [9, 11, 14].

Examining the association of BMI with FHH, some epidemiological studies found that, compared with individuals with no FHH, mean levels of BMI among offspring with a FHH were significantly higher by approximately 0.5 kg/m^2^ to 1.7 kg/m^2^ in western countries [12, 15] and by 0.2 kg/m^2^ to 1.5 kg/m^2^ in Asia [7, 8, 10, 16]. In China, interactions between BMI, FHH, and age were found to influence the risk of hypertension on multiplicative scale, suggesting that genetic and environmental factors could be driving this interaction [17]. However, interaction between BMI and FHH on additive scale seems to have escaped attention despite its large potential impact.

Yugur is a minority ethnicity concentrated in Gansu province, China. Compared with Han people, Yugur people have similar living environment but different heredity and culture that lead to a different diet and lifestyle. The aggravation of familial predisposition for hypertension by overweight or obesity is important for the development of prevention and intervention plans and therefore needs to be taken into consideration. However, the interplay of BMI and FHH has not been well explored in Han and Yugur people. In this study, we examined the associations of BMI with FHH and their interaction with hypertension in different ethnicities and whether these associations and interactions could be influenced by behavioural risk factors.

## 2. Materials and Methods

### 2.1. Study Design and Population

Data for the current cross-sectional study were from the China National Health Survey (CNHS) in Gansu province in 2016 [18], which was conducted by Chinese Academy of Medical Sciences for evaluating the physiological constant and health condition in general Chinese population. According to the protocol, we investigated two ethnicities: Han and Yugur (a representative minority). The sample size of each ethnicity was calculated as follows:

(1) $$\begin{array}{c} N=\frac{Z_{1-\alpha /2}^{2}P\left(1-P\right)}{d^{2}}, \end{array}$$

where* P*=29.6% [5], d=15%×*P*, and *α*=0.05. By performing a multistage stratified clustering sampling method, eligible participants (n=5884) were recruited from Han and Yugur residents aged 20 to 80 years who had been living in Gansu province for more than one year. The sampling process was stratified according to the degree of urbanization, and the final selected four districts were Chengguan District of Lanzhou, Ganzhou District of Zhangye, Yugur Autonomous County of Sunan, and Gaotai County. Data on demographic information, smoking status, alcohol consumption, history of hypertension, FHH, and occupational and leisure-time physical activity were collected face-to-face by well-trained investigators using a uniform questionnaire for all participants. Written informed consent was obtained from every participant before data collection. The study was approved by Institutional Review Board of the Institute of Basic Medical Sciences, Chinese Academy of Medical Sciences.

Participants who had no history of hypertension and BP measurements (n=7) or had missing values on BMI (n=86) were excluded. Following these exclusions, a total of 5791 participants (2499 males and 3292 females) were included in the analyses.

### 2.2. Measures and Procedures

#### 2.2.1. Blood Pressure

Blood pressure was measured 3 times using the Omron digital blood pressure measuring device (HEM907, Japan) on the right arm with participant seated, and the average of the 3 measurements was used as mean blood pressure for analyses. Participants were classified as hypertensive if mean systolic blood pressure (SBP) ≥ 140 mm Hg and/or mean diastolic blood pressure (DBP) ≥ 90 mm Hg and/or in case of physician-diagnosed hypertension and/or self-report of current antihypertensive medication use [19].

#### 2.2.2. Family History of Hypertension

FHH was assessed based on self-report of participant about whether any of his/her family members (living or deceased) had hypertension diagnosed by a physician. Provided options were “yes,” “no,” and “don't know.” If a participant selected “yes,” he/she was asked to ascertain the presence of hypertension in grandfather, grandmother, mother, father, siblings, and offspring. Positive FHH was defined as having at least one family member with hypertension. Thus, those who were unaware (“don't know”) of their family history were regarded as having no FHH. Information regarding offspring was not considered.

#### 2.2.3. Body Mass Index

Height and weight were measured with participants wearing light clothing and without shoes. Height was measured to the nearest 0.1 cm using a fixed stadiometer and weight was measured to the nearest 0.1 kg with a body composition analyser (BC-420, TANITA, Japan). BMI was defined as weight in kilograms divided by the square of height in meters (kg/m^2^). Overweight and obesity were defined as 24.0 kg/m^2^ ≤ BMI<28.0 kg/m^2^ and BMI ≥ 28.0 kg/m^2^, respectively, based on Chinese criteria [20].

#### 2.2.4. Ethnicity

Ethnicity was verified by certificate of identification and categorized as Han and Yugur people. A participant was considered to be Han or Yugur people if he/she and his/her parents were of the same ethnicity.

#### 2.2.5. Health-Related Behaviour

Health-related behaviour was measured by smoking status, alcohol consumption, and physical activity. As described in detail before [18], smoking status was categorized as participants being a nonsmoker, former smoker, or current smoker, and alcohol consumption was categorized as participants being a nondrinker, former drinker, or current drinker. Physical activity included occupational and leisure-time physical activity and was categorized as participants being low, moderate, or high [21].

#### 2.2.6. Statistical Analyses

Continuous variables were presented as mean ± standard deviation (SD) and categorical variables were expressed as n (%), and differences between two groups were examined by Student's* t*-test and Chi-square test with a significance level of 0.05, respectively. All of these and following analyses were performed using SAS software version 9.4 (SAS Institute Inc., Cary, NC, USA).

Associations of BMI and FHH with hypertension were assessed by logistic regression models. Considering the difference between Han and Yugur people, all analyses were stratified by ethnicity. Odds ratios (ORs) with 95% confidence intervals (95%* CIs*) were presented for the combination of each category of BMI and FHH with normal weight and no FHH regarded as a reference category.

As Knol and VanderWeele proposed [22], we examined the associations of BMI and hypertension across strata of FHH (*i.e.*, yes and no) and the associations of FHH and hypertension across strata of BMI (*i.e.*, normal weight, overweight, and obesity). Thus, additional information was offered on the interaction effects by these analyses.

Since the additive model was the basis for assessing biological interaction, we measured interaction of BMI and FHH on additive scale [22–24]. Lundberg M* et al.* recommended a SAS program that was set up to calculate the measures of the interaction with confidence intervals [25]. In order to specify the model, let* i*=1 when overweight was present and 0 when normal weight was present, and let* j*=1 when FHH was present and 0 when FHH was not present. Therefore, obese participants were not included in these analyses but separate analyses were performed. Furthermore, let OR_*ij*_ be the OR in exposure category* i, j*. Thus, with OR_00_ as reference category, there were three ORs (OR_11_, OR_10_, and OR_01_) to be estimated. Rothman presented three measures of biological interaction: RERI, the relative excess risk due to interaction; AP, the attributable proportion due to interaction; and SI, the synergy index [26]. These measures were defined as follows: RERI= OR_11_−OR_10_−OR_01_+1; AP= RERI/OR_11_; SI= (OR_11_−1)/[(OR_01_−1)+(OR_10_−1)].

RERI, AP, and SI were calculated using the regression coefficients and covariance matrix obtained from the multivariate logistic regression analyses [25–27]. The delta method, which Andersson* et al.* [27, 28] referred to, was used to calculate the 95%* CI* of RERI, AP, and SI. If the 95%* CIs* of RERI and AP did not include 0 and the 95%* CI* of SI did not include 1, interaction was present. We repeated all analyses regarding obesity as the risk factor compared with normal weight, with obesity coded as 1 and normal weight as 0.

To explore whether the associations and interactions of BMI and FHH on hypertension could be influenced by behavioural risk factors, the basic logistic regression model on age and gender was further adjusted for behavioural risk factors, including current residence, educational level, smoking status, alcohol consumption, and physical activity.

## 3. Results

Demographic characteristics of the 5791 participants (4195 Han and 1596 Yugur) varying by ethnicity and FHH were shown in Table 1. Within each ethnic group strata, compared with participants who reported a FHH, participants who reported no FHH had lower systolic and diastolic blood pressure and prevalence of hypertension.

**Figure 1:**
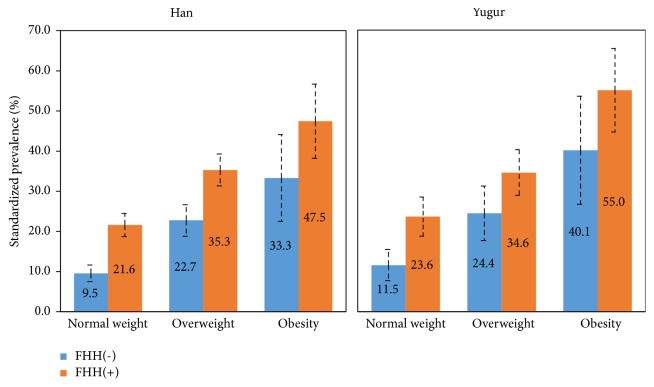
Standardized prevalence of hypertension according to categories of BMI and FHH in Han and Yugur people in China. Prevalence of hypertension was age- and gender-standardized based on 2010 China census population data of Gansu province using direct standardization method. Bars represent proportion and error bars (95%* CI*). BMI: body mass index, FHH: family history of hypertension.

Figure 1 showed the age- and gender-standardized prevalence of hypertension in Han and Yugur people based on 2010 China census population data of Gansu province using direct standardization method. Participants with a FHH had higher prevalence compared with those with no FHH in each BMI category of two ethnicities. As BMI categories increased from normal weight to obesity, the prevalence also increased in both groups with and without a FHH. Across categories of BMI and FHH, the standardized prevalence of hypertension was highest for those with an obese status and a FHH in both Han (47.5%) and Yugur people (55.0%).

**Figure 2:**
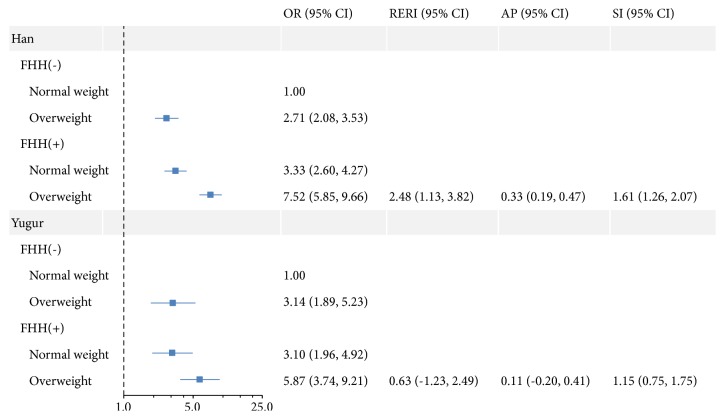
Associations and interactions of overweight and FHH on hypertension in Han and Yugur people in China. Models were adjusted for age and gender. FHH: family history of hypertension, RERI: the relative excess risk due to interaction, AP: the attributable proportion due to interaction, SI: the synergy index. Interaction was present if the 95%* CIs* of RERI and AP did not include 0 and the 95%* CI* of SI did not include 1.

The associations and interactions of overweight and FHH on hypertension were shown in Figure 2. The ORs for hypertension were highest for Han and Yugur people with overweight and a FHH compared with those with normal weight and no FHH (Han: OR: 7.52, 95%* CI*: 5.85 to 9.66; Yugur: OR: 5.87, 95%* CI*: 3.74 to 9.21). A significant interaction between overweight and FHH was observed in Han people, with the RERI, AP, and SI and their 95%* CIs *being 2.48 (1.13 to 3.82), 0.33 (0.19 to 0.47), and 1.61 (1.26 to 2.07), respectively. It suggested that there was a 2.48 relative excess risk due to the additive interaction, and 33% of hypertension due to both overweight and a FHH was attributable to the additive interaction. The interaction was not observed in Yugur people since the 95%* CIs* of RERI and AP included 0 and the 95%* CI* of SI included 1.

**Figure 3:**
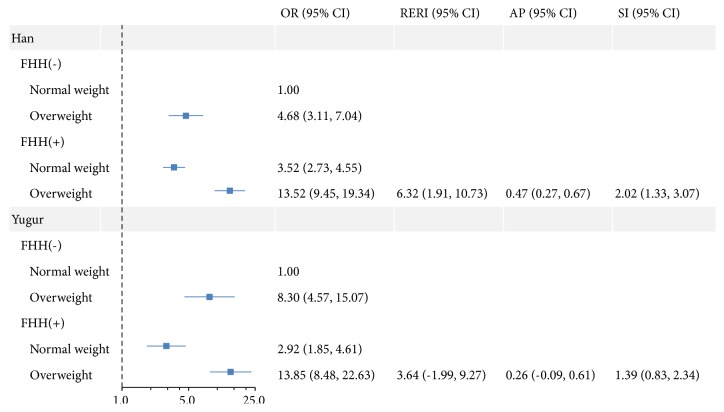
Associations and interactions of obesity and FHH on hypertension in Han and Yugur people in China. Models were adjusted for age and gender. FHH: family history of hypertension, RERI: the relative excess risk due to interaction, AP: the attributable proportion due to interaction, SI: the synergy index. Interaction was present if the 95%* CIs* of RERI and AP did not include 0 and the 95%* CI* of SI did not include 1.

The results of associations and interactions of obesity and FHH on hypertension shown in Figure 3 were similar to Figure 2. The ORs for hypertension were highest for Han and Yugur people with obesity and a FHH compared with those with normal weight and no FHH (Han: OR: 13.52, 95%* CI*: 9.45 to 19.34; Yugur: OR: 13.85, 95%* CI*: 8.48 to 22.63). A significant interaction between obesity and FHH was also only observed in Han people, with the RERI, AP, and SI and their 95%* CIs *being 6.32 (1.91 to 10.73), 0.47 (0.27 to 0.67), and 2.02 (1.33 to 3.07), respectively.

The ethnicity-specific ORs for hypertension in each category of FHH and BMI were shown in Table 2. For both those with and without a FHH, the BMI group gradient in ORs for hypertension was steeper for Yugur than for Han people. Additionally, compared with those having no FHH, participants with a FHH had significant risks for hypertension in all of other BMI groups in two populations except for the obesity group in Yugur people.

After adjusting for age, gender, current residence, educational level, smoking status, alcohol consumption, and physical activity, interaction of overweight (or obesity) and FHH on hypertension was still present in Han people and was still not observed in Yugur people. Since the ORs, RERIs, Aps, and SIs had little change, these behavioural risk factors hardly influenced the associations and interactions, and more than 14 times risks remained in both two populations with obesity and a FHH.

## 4. Discussion

The study showed that BMI and FHH were associated with hypertension and the interaction of BMI and FHH on hypertension was significant in Han but not in Yugur people. Behavioural risk factors had little influence on the associations underlying the interaction between BMI and FHH in two populations.

To the best of our knowledge, this study is the first one to explore the interaction of BMI and FHH on hypertension using an additive model in Gansu province. Few studies had examined the interactions but not on additive scale. The Atherosclerosis Risk in Communities (ARIC) Study, which comprised the population of European-Americans and African-Americans, indicated no effect modification of family history by BMI [29]. However, Eva G. Katz* et al.* found that the interaction between BMI and FHH stratified by age was significant to influence the risk of hypertension in Chinese adults [17].

The finding that BMI and FHH were associated with hypertension is consistent with previous cross-sectional and longitudinal studies [7–9, 11–15, 17]. After adjustment for all of the behavioural risk factors in our study, ORs for hypertension in Han and Yugur people with overweight (or obesity) and a FHH remained significant (Table 3). Other unmeasured risk factors, for instance, hypoadiponectinemia, C-reactive protein, anticipatory stress, and obstructive sleep apnoea syndrome, may play a role in the development of hypertension as well and may further explain these associations and interactions [30–33].

Reports of FHH depend on prevalence of hypertension, availability of diagnostic facilities, health-seeking behaviours, and communication among family members. Most of these factors are likely to be common but different within and between populations. Among Chinese adults, rates of hypertension awareness (42.6%), treatment (34.1%), and control (9.3%) [5] were lower compared with rates of awareness (75.7%), treatment (65.1%), and control (36.8%) in the United States [34]. If only about half of siblings, parents, and grandparents who were hypertensive were aware of their hypertension, the family history status that offspring reported was most likely underestimated in our study. A study of sub-Saharan African in Gambia found that participants who reported a family history of noncommunicable disease were younger, more likely to live in a city, better educated, and more likely to be female [35]. Since awareness of hypertension differs in different time, older participants may be less aware of FHH compared with younger ones.

In the current study, FHH was used to define the predisposition for hypertension, which may also be defined through a genetic risk score (GRS) based on risk loci obtained from genome-wide association studies (GWAS) [36, 37]. FHH contains both the genetic predisposition for hypertension and the shared environment factors within a family. Since it does not allow discrimination between the effects of genes and environment factors, FHH may be a better predictor of hypertension than a GRS. Although both a FHH and a GRS for hypertension assess hypertension predisposition, future studies should examine whether the use of a GRS would lead to different results from the results from this study. Additionally, future studies should focus on epigenetic changes related to obesity as they may contribute to the development of hypertension [38–40].

This study has a number of strengths worth mentioning. Firstly, our study is the first one to estimate interactions of BMI and FHH on hypertension on additive scale in Gansu province. Secondly, due to the interactions estimated on additive scale, we figured out how much relative excess risk was due to the additive interaction and how much of hypertension exposed to both overweight (or obesity) and a FHH was due to the additive interaction.

The present study also has some limitations. Firstly, the nature of cross-sectional study does not allow us to make inference about the causality for the effects. Secondly, it was difficult to verify the facticity of FHH and, therefore, recall and information bias may exist in our study. Thirdly, as we did not test genome-wide genotype data for participants, effects of genes and environment factors cannot be discriminated. Future studies are needed to examine whether the use of a GRS would lead to different results.

## 5. Conclusions

BMI and FHH were associated with hypertension, and the interaction of BMI and FHH on hypertension was significant in Han but not in Yugur people. Behavioural risk factors had little influence on the associations underlying the interaction between BMI and FHH, and more than 14 times risks remained in two populations with obesity and a FHH. The exacerbation of hypertension risks by overweight or obesity in hypertension families deserves attention in weight control and community care.

## Figures and Tables

**Table 1:** Demographic characteristics of 5791 participants by ethnicity and FHH in China. ^a^

	Han (N=4195)			Yugur (N=1596)		
	FHH (-)	FHH (+)	*P*	FHH (-)	FHH (+)	*P*
	(N=1855)	(N=2340)		(N=547)	(N=1049)	
Age (yrs.)	52.30±12.30	48.54±11.66	<0.0001	49.31±12.54	46.99±11.54	0.0003
Gender (%)			0.0085			0.1219
Male	816 (43.99)	935 (39.96)		271 (49.54)	477 (45.47)	
Female	1039 (56.01)	1405 (60.04)		276 (50.46)	572 (54.53)	
BMI (kg/m^2^)	23.69±3.06	23.96±3.16	0.0047	24.48±3.79	25.12±3.94	0.0019
BMI category (%)			0.1036			0.0070
Normal weight	1010 (54.45)	1233 (52.69)		261 (47.71)	420 (40.04)	
Overweight	692 (37.30)	870 (37.18)		194 (35.47)	402 (38.32)	
Obesity	153 (8.25)	237 (10.13)		92 (16.82)	227 (21.64)	
SBP (mm Hg)	119.47±16.08	121.73±17.38	<0.0001	122.19±15.02	123.69±16.63	0.0679
DBP (mm Hg)	72.96±10.26	75.49±11.28	<0.0001	75.88±10.58	77.82±11.66	0.0008
Hypertension (%)			<0.0001			<0.0001
Yes	387 (20.86)	783 (33.46)		142 (25.96)	387 (36.89)	
No	1468 (79.14)	1557 (66.54)		405 (74.04)	662 (63.11)	
Current residence (%)			<0.0001			0.0059
Urban	1121 (60.43)	1705 (72.86)		175 (31.99)	409 (38.99)	
Rural	734 (39.57)	635 (27.14)		372 (68.01)	640 (61.01)	
Educational level (%) ^b^			<0.0001			0.0132
Low	707 (38.20)	530 (22.72)		310 (56.67)	559 (53.49)	
Medium	735 (39.71)	1002 (42.95)		176 (32.18)	312 (29.86)	
High	409 (22.10)	801 (34.33)		61 (11.15)	174 (16.65)	
Smoking status (%) ^c^			0.0002			0.8351
Former smoker	209 (11.27)	179 (7.65)		54 (9.88)	106 (10.10)	
Current smoker	400 (21.56)	494 (21.12)		168 (30.71)	307 (29.27)	
Non-smoker	1246 (67.17)	1666 (71.23)		325 (59.41)	636 (60.63)	
Alcohol consumption (%) ^d^			0.0001			0.4198
Former drinker	212 (11.44)	228 (9.74)		81 (14.81)	177 (16.87)	
Current drinker	798 (43.07)	1162 (49.66)		292 (53.38)	565 (53.86)	
Nondrinker	843 (45.49)	950 (40.60)		174 (31.81)	307 (29.27)	
Physical activity (%) ^e^			0.0211			0.3711
Low	249 (13.44)	345 (14.74)		54 (9.87)	105 (10.02)	
Moderate	1319 (71.22)	1703 (72.78)		366 (66.91)	667 (63.65)	
High	284 (15.33)	292 (12.48)		127 (23.22)	276 (26.34)	

^a^Data were shown as Mean ± SD or N (%). FHH: family history of hypertension, BMI: body mass index, SBP: systolic blood pressure, DBP: diastolic blood pressure. The amount of missing data:  ^b^educational level: 15,  ^c^smoking status: 1,  ^d^alcohol consumption: 2,  ^e^physical activity: 4.

**Table 2:** Associations of BMI with hypertension per category of FHH and associations of FHH with hypertension per category of BMI in Han and Yugur people in China.

	Han		Yugur	
	OR (95% *CI*)	*P*	OR (95% *CI*)	*P*
**The association of BMI and hypertension per category of FHH**				
*FHH(-)*				
Normal weight	1.00		1.00	
Overweight	2.70 (2.08, 3.52)	<0.0001	3.20 (1.92, 5.42)	<0.0001
Obesity	4.33 (2.89, 6.47)	<0.0001	8.55 (4.65, 16.10)	<0.0001
*FHH(+)*				
Normal weight	1.00		1.00	
Overweight	2.22 (1.80, 2.75)	<0.0001	1.91 (1.36, 2.70)	0.0002
Obesity	3.76 (2.73, 5.19)	<0.0001	5.04 (3.42, 7.50)	<0.0001
**The association of FHH and hypertension per category of BMI**				
*Normal weight*				
FHH(-)	1.00		1.00	
FHH(+)	3.84 (2.94, 5.04)	<0.0001	2.72 (1.73, 4.38)	<0.0001
*Overweight*				
FHH(-)	1.00		1.00	
FHH(+)	2.69 (2.12, 3.43)	<0.0001	1.93 (1.26, 2.99)	0.0028
*Obesity*				
FHH(-)	1.00		1.00	
FHH(+)	2.90 (1.81, 4.73)	<0.0001	1.76 (1.00, 3.12)	0.0525

Models were adjusted for age, gender, current residence, educational level, smoking status, alcohol consumption, and physical activity.

BMI: body mass index, FHH: family history of hypertension.

**Table 3:** Associations and interactions of BMI and FHH on hypertension in Han and Yugur people adjusted for behavioral risk factors in China.^a^

	Han (N=4195)				Yugur (N=1596)			
	OR (95% *CI*)	RERI (95% *CI*)	AP (95% *CI*)	SI (95% *CI*)	OR (95% *CI*)	RERI (95% *CI*)	AP (95% *CI*)	SI (95% *CI*)
**Model overweight**								
*FHH*(-)								
Normal weight	1.00				1.00			
Overweight	2.77 (2.12, 3.61)				3.15 (1.89, 5.25)			
*FHH*(+)								
Normal weight	3.54 (2.75, 4.57)				2.96 (1.86, 4.71)			
Overweight	7.91 (6.12, 10.23)	2.60 (1.17, 4.03)	0.33 (0.19, 0.47)	1.60 (1.26, 2.05)	5.74 (3.65, 9.03)	0.63 (-1.22, 2.48)	0.11 (-0.20, 0.42)	1.15 (0.75, 1.77)
**Model obesity**								
*FHH*(-)								
Normal weight	1.00				1.00			
Obesity	4.61 (3.05, 6.98)				8.09 (4.44, 14.77)			
*FHH*(+)								
Normal weight	3.74 (2.87, 4.86)				2.81 (1.77, 4.45)			
Obesity	14.06 (9.76, 20.25)	6.72 (2.10, 11.33)	0.48 (0.28, 0.67)	2.06 (1.36, 3.11)	14.16 (8.61, 23.28)	4.25 (-1.56, 10.06)	0.30 (-0.04, 0.64)	1.48 (0.87, 2.51)

^a^Models were adjusted for age, gender, current residence, educational level, smoking status, alcohol consumption, and physical activity.

BMI: body mass index, FHH: family history of hypertension, RERI: the relative excess risk due to interaction, AP: the attributable proportion due to interaction, SI: the synergy index.

Interaction was present if the 95% *CIs* of RERI and AP did not include 0 and the 95% *CIs* of SI did not include 1.

## Data Availability

An application for the original data can be provided via http://www.bmicc.cn/web/share/home.
